# Comparative Studies on the Ecophysiological Differences of Two Green Tide Macroalgae under Controlled Laboratory Conditions

**DOI:** 10.1371/journal.pone.0038245

**Published:** 2012-08-08

**Authors:** Ying Wang, You Wang, Lin Zhu, Bin Zhou, Xuexi Tang

**Affiliations:** Department of Marine Ecology, College of Marine Life Sciences, Ocean University of China, Qingdao, China; University of New South Wales, Australia

## Abstract

Yellow Sea green tides have occurred in coastal China almost every year from 2007 to 2011. *Ulva prolifera* (Müller) J. Agardh has been identified as the causative macroalgal species. *U. intestinalis*, however, has been observed in the bloom areas, co-occurring with *U. prolifera*, but it has not been found to be causative. The Yellow Sea green tide has shown consistent phases of development that match corresponding environmental changes. *U. prolifera*, not *U. intestinalis*, is dominant. Our experimental design was based on these observed phenomena, and the results of our field investigation indicated a close relationship between changes in principal environmental factors (irradiance, temperature, and salinity) and the development of each phase of the bloom. These main environmental factors were simulated to allow estimation and comparison of the physiological responses of *U. prolifera* and *U. intestinalis*. Ecophysiological differences were found between these two species. (1) More photosynthetic activity and plasticity were detected in *U. prolifera*. (2) *U. prolifera* was found to be more sensitive to dynamic environments, especially harsh and changing environmental conditions. *U. intestinalis* was found to be more stable, probably due to the higher stress tolerance given by its antioxidant system. (3) Markedly higher nutrient absorption activity was observed in *U. prolifera*. Comparisons of the ecophysiological traits of these two species in this present study may foster understanding of their natural ecological processes. Specifically, *U. prolifera* seemed to be more engaged with the ephemeral blooms, while *U. intestinalis* seemed to be directed toward persistence. This also suggests that the ecological success of *U. prolifera* may be inextricably linked to its higher capacity for photosynthesis, nutrient absorption, and nutrient assimilation.

## Introduction

Over the past decade, the excessive growth of some species of green algae, mainly within the genus *Ulva*, has been reported as cause of green tides in many parts of the world, including Europe, the Americas, Australia, and Asia [1], [2], [3], [4], [5], [6], [7], [8], [9]. Typically, green tides are characterized by the choking of navigation channels in the immediate area of the bloom and local deposition on the shore. This can be destructive to estuary communities at different trophic levels. It also causes economic losses to fisheries and tourism [10], [11].

Green tides are related to a series of complex coupled processes and various biotic and abiotic factors are thought to affect the spatial patterns of green tides [1], [5], [6]. In case studies of *Ulva* species, the most relevant environmental factors were found to include irradiance, temperature and salinity [12], [13], [14], [15]. Photosystem II (PS II) activity and antioxidant system performance in these *Ulva* species were sensitive to environmental stress conditions, so previous studies used them as indicators of physiological response states. However, they have only rarely been used to explain dominance among groups of species, especially co-occurring species. The environmental factors connected to the diminishment of green tides after the bloom period are not well understood. We therefore speculated that the dominant species gains a competitive advantage by exhibiting either pronounced adaptability to wide ranges of irradiance, temperature, and salinity or physiological adaptations to variable environmental conditions.

Large-scale green tides in China's Yellow Sea, called the “Yellow Sea green tide,” have broken out continuously from 2007 to 2011. The first green tide occurred off the coast of Qingdao in late July, 2007 [7]. A much larger one was observed from May to July of 2008. Because Qingdao was one of the host cities of the Olympic Games in 2008, the green tide posed a seriously threat to the regatta race. This brought worldwide attention to green tides. In May–August of 2009, 2010, and 2011, similarly large green tides bloomed in the same sea area [15], [16]. This event proved regular, recurring in each of the last five years.

The dominant species of the Yellow Sea green tide was found to be *Ulva prolifera (*Müller) J. Agardh [7], [17]. Recent phylogenetic analysis have indicated that it may belong to a unique strain of the *U. linza-procera-prolifera* (LPP) clade [18], [19]. Another green tide macroalgae observed in the present study was *U. intestinalis*, which has been noted worldwide as a green tide-forming species, especially in eutrophic estuaries in Europe and North America [20], [21]. However, it was not found to be the causative species of the green tides observed off the coast of China. It showed different natural ecological processes from those of *U. prolifera*. The *U. intestinalis* population remained attached throughout a year (personal observation), while the annual *U. prolifera* cycle involved a relative ephemeral bloom lasting from May to July followed by a drop in population in August. However, both species were always found to co-occur [7], [15], [16], [22]. Although a number of studies, including remote sensing studies, shipboard surveys, field experiments, and laboratory experiments, have been performed to determine the possible ecological bases of green tides, they have focused on *U. prolifera*. Little attention has been paid to ecological and physiological differences between these co-occurring species.

In the light of their co-occurrence and different natural ecological processes, we chose these two species of green macroalgae for the present study. We decided to assess (1) the relationship between the environmental factors and the bloom-forming period and (2) possible ecological and physiological mechanisms underlying the dominance of *U. prolifera* in the green tides occurring off the coast of Qingdao.

## Materials and Methods

### 1. Site descriptions

The field observations were carried out along the rocky intertidal shores around Taiping Cape (36.0492′N, 120.3536′E), Qingdao, P.R. China. Green tides have broken out in this area during each of the past five years. Many natural macroalgal assemblages were found in these intertidal zones, both attached and free-floating. Within the genus *Ulva*, free-floating *U. prolifera* is the dominant species. It often co-occurs with attached *U. intestinalis* during the green tide period. Our study field, located around Taiping Cape (36.0492`N, 120.3536`E), is frequently studied by the Ocean University of China (OUC) and we had permission from OUC to conduct our experiments. The area is a seaside resort open to the public, and no endangered species live there.

### 2. Macroalgal cultures

The thalli of *U. prolifera* and *U. intestinalis* were collected from coastal Qingdao in June 2010 during the bloom period. The thalli were rinsed gently in sterile seawater and cleaned thoroughly with a brush under a magnifier to remove the attached sediment, small grazers, and epiphytes. They were then cultured in sterile seawater enriched with f/2 medium at a constant temperature of 20°C and light intensity of 72 μmol photons.m^−2^s^−1^ in a 12:12 h light:dark cycle in a GXZ-280 C intelligent illumination incubator (Ningbo Jiangnan Instrument, China) for acclimation before of experiments [23]. Germanium dioxide (GeO_2_) at a concentration of 0.5 mg l^−1^ was added to the cultures to suppress diatom growth [24]. The culture medium was completely renewed every two days. The following experiments were performed under to the same conditions using the procedures described above except where otherwise stated.

### 3. Field investigation and experimental design

We divided the green tide bloom into three periods according to the field observations: The blooming period from May to July was designated “*bloom*.” The period during which the bloom diminished, from August to September, was designated “*post-bloom*.” All other months were designated “*pre-bloom*.” The environmental factors were analyzed simultaneously within the green tide. The aim of this part of the study was to determine whether these environmental factors varied along with the phases of the green tide.

The environmental factors measured in the present study mainly included photosynthetically active radiation (abbreviated “PAR” in the present study), surface seawater temperature (abbreviated “SST”), surface seawater salinity (abbreviated “SSS”), and dissolved inorganic nitrogen (abbreviated “DIN”). They were investigated from November 2009 to March 2011 at ebb tides. SST and SSS were measured with a T-S meter (YSI 30; Omni Controls Inc, U.S.). Seawater samples for nutrient analysis were filtered through GF/C filters (Whatman, U.S.) and stored -80°C. DIN (nitrate, nitrite, and ammonium) concentrations were measured using standard procedures. Light intensity was recorded as total luminous flux (lm.m^−2^) from March 2010 to March 2011 (about three times per month). We transformed monthly means of total luminous flux to PAR (400–700 nm, μmol photons.m^−2^.s^−1^) according to the method described by Thimijan [25].

The number of typhoons (mature tropical cyclones) capable of affecting China's offshore regions and total rainfall was recorded from January 2010 to December 2010. Data were obtained from the World Weather Information Service Organization database (http://www.worldweather.org/).

The relationship between the environmental factors and the phases of the bloom was determined using statistical methods. When any relationship was detected between them, the experimental design was carried out using environmental factors during different phases. Otherwise, a full factorial experiment was designed in the laboratory experiments.

### 4. Laboratory experiments

#### 4.1. Experimental set-up

The laboratory experiments focused on the physiological responses of these two species upon exposure to different environmental factors. These factors were simulated using data from field investigations. The treatments were divided into three groups:

1. *pre-bloom*, at 10°C, 30 μmol photons.m^−2^.s^−1^,35 practical salinity units (PSU);
2. *bloom*, at 20°C, 60 μmol photons.m^−2^.s^−1^, 30 PSU;
3. *post-bloom*, at 26°C, 90μmol photons.m^−2^.s^−1^, 25 PSU.

After acclimation, the thalli were randomly selected and cultured in separate 500 ml glass beakers with five duplicates. They were then transferred into beakers under the conditions given above. The culture experiments were repeated five times. Each lasted 7 days, covering two phases: short-term (24 hours) and long-term (7 days). Salinity was adjusted using NaCl and deionized water. The culture medium was renewed every 2 days to prevent increases in salinity and depletion of nutrients.

#### 4.2. Photosynthesis analysis

The treated thalli were collected at 24 h and 7 d. Various factors related to photosynthesis, including photosynthetic pigments, optimal photochemical efficiency (Fv/Fm, Fv is the variable fluorescence, Fm is the maximal fluorescence), and rapid light respond curves (RLCs) were analyzed.

Samples (0.5 g FW, fresh weight thallus) were ground in liquid nitrogen and extracted using 90% (V/V) acetone buffer (5 ml) and some grit to extract liposoluble pigments. The acquired mixture was then treated with 10,000 rpm at room temperature for 10 min and the supernatant was used for further analyses. The chlorophyll contents were determined spectrophotometrically with a Hitachi F-4500 Fluorescence Spectrophotometer (HITACHI, Japan) within a scanning absorption spectrum (350–700 nm), and the contents were determined using to the following formula:


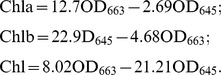


All assays were performed in 5 replicates and the results are expressed in milligrams per gram of fresh weight (mg/g FW).

Photosynthetic efficiency was determined using a portable pulse amplitude modulated (PAM) fluorometer (Mini PAM, Walz, Germany). Samples were dark-adapted under the respective culture treatments for about 20 min before measurement. The Fv/Fm was determined in 5 replicates. No step was repeated on the same tissue sample during the same time period. Then RLCs were measured. The samples were exposed to a light intensity gradient (PAR 0, 24, 38, 55, 81, 122, 183, 262, and 367 μmol photons.m^−2^.s^−1^). Each step lasted 10 s. Then, the relative electron transport rate (rETR) and effective photosynthetic yield (Y(II)) of photosystem II (PSII) values under each light intensity were measured. The RLCs were fitted using the empirical equation determined by Platt et al. in order to determine parameters α (photosynthetic rate in light-limited region of RLC), rETRmax (maximum relative electron transport rate), and Ek (minimum saturating irradiance) [26]. The general protocol for the determination of chlorophyll fluorescence parameters was performed as described by Pang et al. and in the operation handbook from Heinz Walz GmbH (2007).

#### 4.3. Biochemical analysis

On the 7^th^ day after treatment, the thalli were collected, and changes in lipid peroxidation, total antioxidant ability (T-AOC), antioxidant enzymatic activity, and non-enzyme antioxidant activity were analyzed. The activity of the enzyme nitrate reductase (NR), which has a close relationship with nutrient absorption, was estimated simultaneously. Samples (0.2–0.3 g) were ground in liquid nitrogen and extracted using 1.5 ml of 50 mmol/l potassium phosphate buffer (pH7.0) containing 0.1 mmol/l EDTA (Na_2_ ethylenediaminetetraacetic acid). Extracts were centrifuged at 4000 rpm for 15 min and a crude enzymatic extraction was used for lipid peroxidation, antioxidant enzymatic activity, and non-enzymatic antioxidant assay.

As an indicator of oxidative stress, lipid peroxidation was studied as malondialdehyde (MDA) formation. The MDA content was measured using thiobarbituric acid (TBA). Then 0.2 ml of extract was homogenized in 1 ml of 10% trichloroacetic acid (TCA). Homogenates were centrifuged at 4000 rpm and 4°C for 10 min. To each 2 ml aliquot of the supernatant, 2 ml of 0.6% TBA was added. The mixtures were heated at 95°C for 40 min and then quickly cooled in an ice bath. After centrifugation at 4000 rpm and 4°C for 10 min, the absorbance of the supernatant was recorded at 532 nm. Results are expressed as n mol MDA per mg of total soluble protein (n mol MDA/mg pro).

Total levels of soluble protein per g of fresh-weight thallus (TSP, g/g FW) and total levels of soluble protein of the crude extract for antioxidant enzyme activities (protein g/ml, pro. g/ml) were determined using a commercial protein assay (BioRad, U.S.). Protein content was determined spectrophotometrically at 595 nm and concentrations were calculated relative to bovine serum albumin as a standard (Sigma, U.S.). Total antioxidant ability (T-AOC) was determined using a commercial assay kit (Jiancheng Biotech Int., Nanjing, China).

SOD was measured using the xanthine oxidase-cytochrome c reduction method [27]. One unit of SOD was defined as the amount of enzyme required for 50% inhibition of cytochrome c reduction. Glutathione peroxidase (Gpx) activity was measured in a coupled enzyme system where the formed oxidized glutathione (GSSG) was converted to its reduced form by glutathione reductase [28]. Gpx activity was calculated from the initial rate of the reaction after subtracting the non-enzymatic oxidation using the extinction coefficient of 5, 5′-dithiobis (2-nitrobenzoate) (DTNB). One unit of Gpx was defined as the quantity of Gpx required to produce a 1μmol decrease in the GSH concentration per minute. Enzyme activities are expressed as units per mg of total soluble protein (U/mg pro).

Total glutathione (GSH, reduced and oxidized) content was measured using 5, 5′-dithio-bis (2-nitrobenzoic acid) (DTNB). Half-milliliter quantities of extract were extracted with 2 ml of 5% (v/v) 5-sulfosalicylic acid containing 1% (w/v) polyvinylpyrrolidone 40. Homogenates were centrifuged at 4000 rpm at 4°C for 10min. One milliliter of supernatant was mixed with 100 μl of 6 mM DTNB, 175 μl distilled water, and 25 μl of 266 U/ml glutathione reductase. The rates of increase in absorbance at 420 nm were quantified using a standard curve. Enzyme activities were expressed as mg GSH per mg of total soluble protein (mg GSH/mg pro.)

The activity of nitrate reductase (NR) was estimated using the assay method described by Young et al. [29]. The sample was incubated at 20°C and the reaction terminated by the addition of 1 mol/l zinc acetate. The rate of increase (U) in absorbance at 540 nm was estimated by linear regression of increasing NO_2_ concentration over time. Enzyme activities are expressed as units per mg of mg fresh weight thallus (U/mg).

#### 4.4. Statistical analysis

The environmental parameters were tested using a one-way ANOVA with the date as a fixed factor (factor “month”); laboratorialdata were tested using a two-way ANOVA with culture treatment (factor “developing phase”), and species (factor “species”) were treated as fixed factors. All values cited in this paper were obtained from fully independent samples. Data were initially examined with Levene's test for homogeneity and the Shapiro-Wilk test for normality. The Student-Newman-Keuls post-hoc multiple comparison test and Duncan post-hoc test were used if ANOVA indicated a significant effect. Differences between treatment means were considered significant at α<0.05. Data in the present study were analyzed using IBM SPSS Statistics 19 (SPSS Inc, U.S.)

## Results

### 1. Field investigation of environmental factors

**Figure 1:**
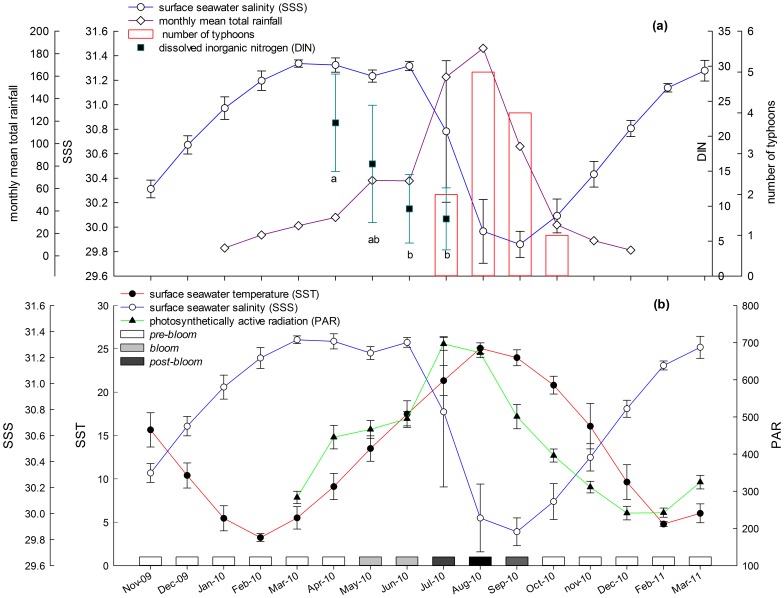
In-site investigation of (a) surface seawater salinity (SSS, PSU), mean total rainfall (mm), number of typhoons, and dissolved inorganic nitrogen (DIN, μmol /L); (b) surface seawater temperature (SST, °C), surface seawater salinity (SSS, PSU), and photosynthetically active radiation (PAR, μmol photons.m^−2^.s^−1^). Total rainfall and number of typhoons were recorded from January 2010 to December 2010. Dissolved inorganic nitrogen (μmol/L) was recorded five times a month from April 2010 to July 2010. Temperature and salinity were measured every week from November 2009 to March 2011. PAR was recorded three times a month from March 2010 to March 2011. Calculated monthly means±standard deviations (SD) are shown. Three different groups of months related to the developmental phases of green tide (*pre-bloom*, *bloom*, and *post-bloom*) are shown as bars. Different color bars and letters indicate significantly different values (*P*<0.05) as determined by post-hoc multiple comparison testing.

Year-round variations in SST, SSS, and PAR from November 2009 to March 2011 are shown in Fig. 1b. An obvious tendency was observed in the changes in SST, which increased from February to August and reached its maximum of 25.8°C in August, after which it decreased steadily from August to the following February. The lowest SST levels were observed in February at 2.7°C. From August to September, the water temperature in the sampling area remained above 24°C. The same tendency was observed in the changes in PAR, which showed a maximum during early July (monthly mean of 697 μmol photons.m^−2^.s^−1^), and a minimum at the end of December (monthly mean of 241 μmol photons.m^−2^.s^−1^). SSS ranged from 28.9–31.4PSU and averaged at 30.7PSU, but its tendencies were quite different from those of temperature and irradiance; the lowest values were observed in September and the highest in March.

**Figure 2.**
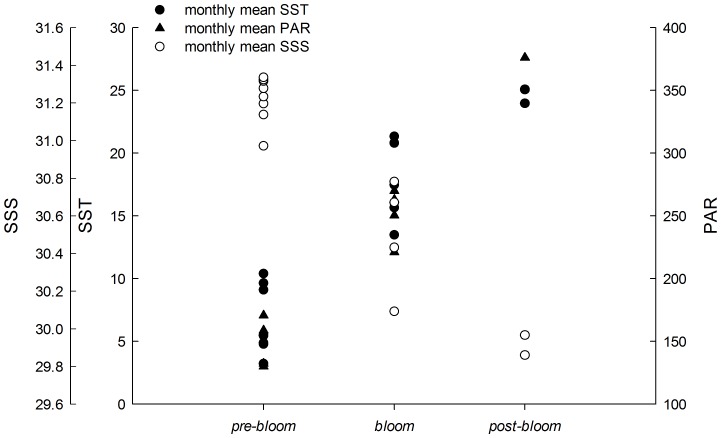
Relationship between green tide phases (*pre-bloom*, *bloom*, and *post-bloom*) and environmental factors (SST-surface seawater temperature-°C, SSS-surface seawater salinity-PSU and PAR-photosynthetically active radiation-μmol photons.m^−2^.s^−1^) from November 2009 to March 2011 offshore of Qingdao.

SST, SSS, and PAR varied quite obviously in different months, and the results of post-hoc multiple comparison tests showed significance between months: SST (F_16, 85_ = 128.3, *P*<0.0005), SSS (F_16, 85_ = 26.1, *P*<0.0005), and PAR (F_11, 36_ = 52.6, *P*<0.0005). Changes in these three factors appeared very regular during different months (Fig. 1b, 2). Mean SST was highest from August to September and lowest during from December to the following April. Means PAR was highest in July and August and lowest from November to the following March. Mean SSS was highest from January to July and lowest in August and September. Three significantly different groups of months were compartmentalized by post-hoc multiple comparison tests: May–late July, late July–early September, and all other months (Fig. 1b).

Field investigations showed an obvious relationship between environmental changes, month, and the phase of the green tide (Fig. 2):

- The bloom period lasted from May to late July and intermediate values of SST, PAR, and SSS were observed. We called this period *bloom*.
- The diminishing period lasted from late July to early September and had high values of SST and PAR but low values of SSS. We called this period *post-bloom*.
- The other months were the preparing period. Low values of SST and PAR and high values of SSS were observed. We called this period *pre-bloom*.

Monthly mean rainfall in 2010 varied considerably from month to month and was negatively associated with salinity (Fig. 1a). Monthly mean rainfall varied from 5.2 mm (December 2010) to 184.8 mm (August 2010) (Fig. 1a). From October to the following April, the monthly mean rainfall did not normally exceed 30mm. From April to June (just before and during the Yellow Sea green tide), monthly mean rainfall showed a small increase. Then, a sudden and sharp increase took place from July to September (during the diminishing phase of Yellow Sea green tide), when more than 62% of total annual rainfall occurred. During the same time, from mid-July to September, 14 out of the year's 15 typhoons took place (Fig. 1a).

Dissolved inorganic nitrogen (DIN) concentrations in the water column ranged from 21.9 to 8.2 μmol/L from April to July, 2010. There were no highly significant differences in DIN concentrations between months (F_3,16_ = 4.905, *P* = 0.013>0.01), but values were found to decrease over time. After post-hoc multiple comparison tests, DIN concentrations from April–May were found to be significantly higher than that from June–July (Fig. 1a). This showed a correlation with rainfall.

### 2. Analysis of photosynthesis

Results of the fluorescence scans showed that either the chlorophyll content or the absorption spectra of the extracted pigments of *U. prolifera* and *U. intestinalis* exposed to different culture treatments were similar. The maximum absorption spectra of chlorophyll a occurred at 436 nm and 663 nm, and the maximum absorption spectra chlorophyll b occurred at 463 and 645 nm.

Pigment concentrations were significantly different between the two species (two-way ANOVA, for all chlorophylls, *P*<0.0005), but the difference among the treated groups was not obvious, especially in the long-term (7days) experiments (two-way ANOVA, for chlorophyll a and total chlorophyll: *P*>0.01) (Table 1). The chlorophyll concentrations in *U. prolifera* were much higher than in *U. intestinalis* in the long-term experiments (Table 1, two-way-ANOVA, *P*<0.0005), especially under the *bloom* culture treatment (Table 2, post-hoc, *P*<0.05). The concentration of chlorophyll b was more affected by experimental treatments than that of chlorophyll a in both the short-term and long-term scenarios (Table 1). However, no significant differences were observed between the ratios of chlorophyll a and b and environmental changes.

**Table 1:** Results of two-way ANOVA on effects of culture treatments (developing phase) and species (species) on the concentration of photosynthetic pigments.

			24h		7d	
Source	Dependent Variable	*df*	F*-*ratio	*P-*value	F*-*ratio	*P-*value
developing phase	Chla	2	16.776	0.000	2.287	0.123
	Chlb	2	20.685	0.000	5.845	0.009
	Chl	2	18.028	0.000	4.153	0.028
	Cha:Chlb	2	12.098	0.000	3.315	0.054
species	Chla	1	30.593	0.000	123.731	0.000
	Chlb	1	5.433	0.028	53.628	0.000
	Chl	1	17.825	0.000	112.554	0.000
	Cha:Chlb	1	0.354	0.557	0.308	0.584
developing phase × species	Chla	2	0.711	0.501	9.581	0.001
	Chlb	2	0.078	0.926	9.720	0.001
	Chl	2	0.039	0.962	9.639	0.001
	Cha:Chlb	2	2.567	0.098	6.038	0.008
Error	Chla	24				
	Chlb	24				
	Chl	24				
	Cha:Chlb	24				

Corresponding pigment concentrations and post-hoc comparisons are shown in Table2.

**Table 2:** Mean concentration of photosynthetic pigments (n = 5) in *U. prolifera* and *U. intestinalis*, measured under different culture treatments: *pre-bloom*, *bloom*, and *post-bloom*.

Treatment	Chlorophyll a		Chlorophyll b		Chlorophyll	
Species	24 h	7 d	24 h	7 d	24 h	7 d
***pre-bloom***						
*U. prolifera*	0.352^a^ ±0.04	0.346^a^ ±0.061	0.157^a^ ±0.058	0.142^a^ ±0.006	0.479^a^ ±0.072	0.501^a^ ±0.058
*U. intestinalis*	0.217^bc^ ±0.07	0.199^c^ ±0.054	0.128^ab^ ±0.039	0.125^a^ ±0.037	0.354^b^ ±0.354	0.333^b^ ±0.091
***bloom***						
*U. prolifera*	0.261^b^ ±0.009	0.356^a^ ±0.019	0.085^bc^ ±0.007	0.153^a^ ±0.022	0.355^b^ ±0.017	0.522^a^ ±0.043
*U. intestinalis*	0.169^cd^ ±0.043	0.115^d^ 0.013	0.051^c^ ±0.017	0.044^c^ ±0.018	0.226^cd^ ±0.055	0.163^c^ ±0.031
***post-bloom***						
*U. prolifera*	0.194^bc^ ±0.056	0.294^b^ ±0.034	0.062^c^ ±0.022	0.141^a^ ±0.022	0.262^bc^ ±0.08	0.446^a^ ±0.059
*U. intestinalis*	0.109^d^ ±0.066	0.206^c^ ±0.028	0.04^c^ ±0.032	0.082^b^ ±0.023	0.152^d^ ±0.099	0.294^b^ ±0.052

Samples were collected after short-term (24 h) and long-term (7 d) culture. Values are means (±SD). Data are mg/fresh weight (g) (mg/g FW) for each pigment, and different superscripts indicate significantly different values (*P*<0.05), as determined by post-hoc comparison.

**Figure 3.**
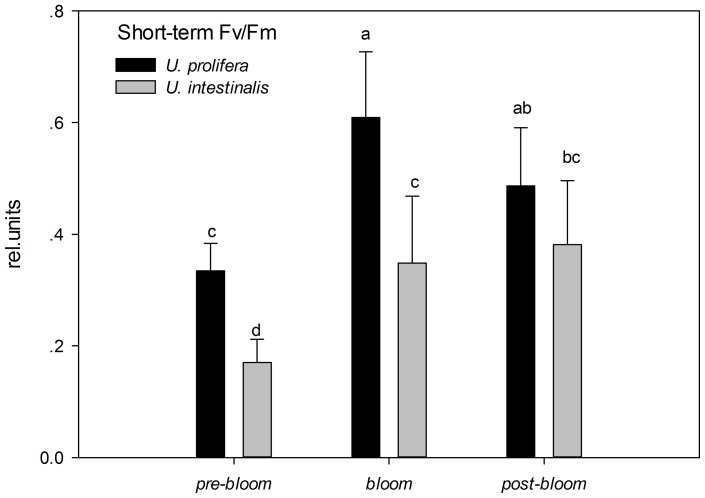
Mean optimal photochemical efficiency of photosynthesis (Fv/Fm) of *U. prolifera* and *U. intestinalis*, obtained from short-term (24 h) culture to each, measured under different culture treatments: *pre-bloom*, *bloom*, and *post-bloom*. Values are means ± SD (n = 5). Different letters above the bars indicate significantly different values (post-hoc, *P*<0.05)

**Figure 4.**
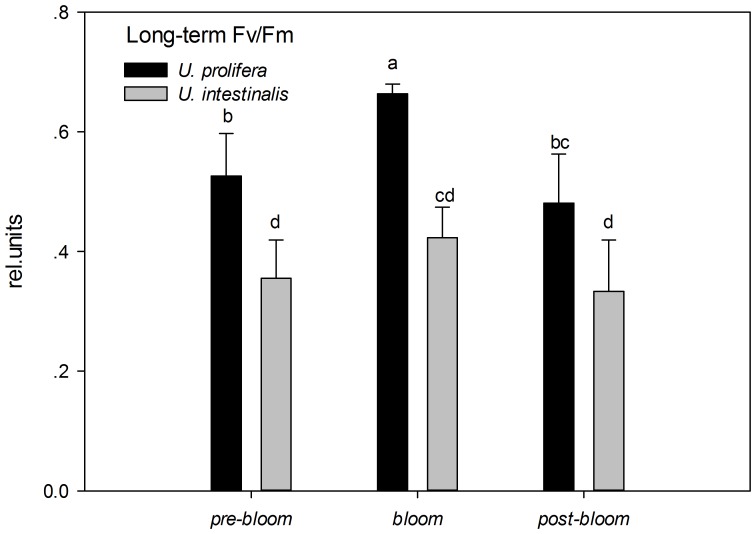
Mean optimal photochemical efficiency of photosynthesis (Fv/Fm) of *U. prolifera* and *U. intestinalis*, obtained from long-term (7 d) culture to each, measured under different culture treatments: *pre-bloom*, *bloom*, and *post-bloom*. Values are means ± SD (n = 5). Different letters above the bars indicate significantly different values (post-hoc, *P*<0.05).

Fv/Fm is a sensitive index that can indicate a plant's photosynthetic status. In the present study, Fv/Fm showed significant and pronounced species and phase effects in both short- and long-term experiments (Table 3, two-way ANOVA, *P*<0.0005). The Fv/Fm of *U. prolifera* was significantly higher than that of *U. intestinalis*, especially during the *bloom* culture treatment (Figs. 3 and 4, post-hoc, *P<0.05*). The maximum initial values of Fv/Fm occurred under *bloom* conditions in both the short- and long-term experiments. For *U. intestinalis*, this decrease was observed when it was exposed to *pre-bloom* culture treatment in the short-term experiments, but results of other treatments showed little change (Figs. 3 and 4, post-hoc, *P*>0.05). In addition, both species showed higher Fv/Fm values in long-term experiments than in short-term experiments under the *pre-bloom* and *bloom* conditions, but almost no changes were observed under *post-bloom* conditions (Figs. 3 and 4).

**Table 3:** Results of two-way ANOVA on effects of culture treatments (developing phase) and species (species) on the optimal photochemical efficiency of photosystem II (Fv/Fm), maximum effective photosynthetic yield (maximum Y (II)), and maximum relative electron transport rate (rETRmax) after short-term (24 h) and long-term (7 d) culture.

			short-term		long-term	
Source	Dependent Variable	*df*	F*-*ratio	*P-*value	F*-*ratio	*P-*value
developing phase	Fv/Fm	2	15.250	.000	11.572	.000
	maximum Y (II)	2	2.602	.095	2.117	.142
	rETRmax	2	31.416	.000	13.089	.000
species	Fv/Fm	1	24.912	.000	59.826	.000
	maximum Y (II)	1	11.771	.002	42.340	.000
	rETRmax	1	133.358	.000	188.005	.000
developing phase × species	Fv/Fm	2	1.647	.214	1.354	.277
	maximum Y (II)	2	.646	.533	.273	.764
	rETRmax	2	16.908	.000	.975	.392
Error	Fv/Fm	24				
	maximum Y (II)	24				
	rETRmax	24				

**Figure 5:**
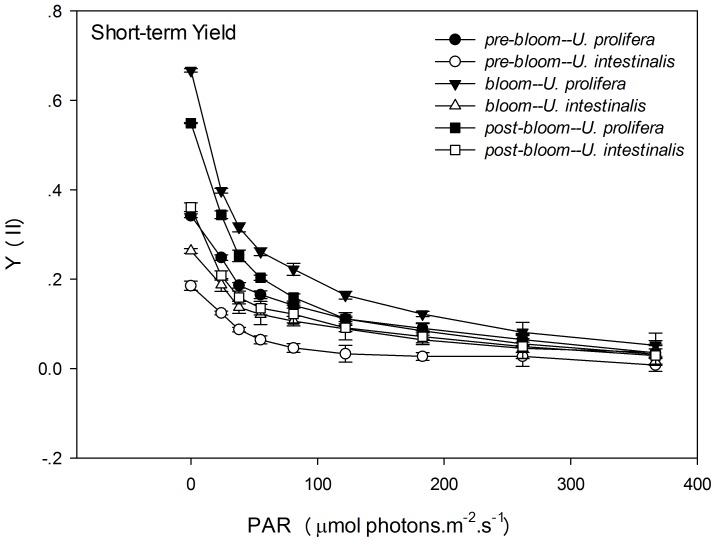
Mean effective quantum yield (Y (II)) of rapid light response curves (RLCs) of *U. prolifera* and *U. intestinalis*, obtained from short-term (24 h) culture to each, measured under different culture treatments: *pre-bloom*, *bloom*,and *post-bloom*. Values are means ± SD (n = 5). Different letters above bars indicate significantly different values (post-hoc, *P*<0.05).

**Figure 6:**
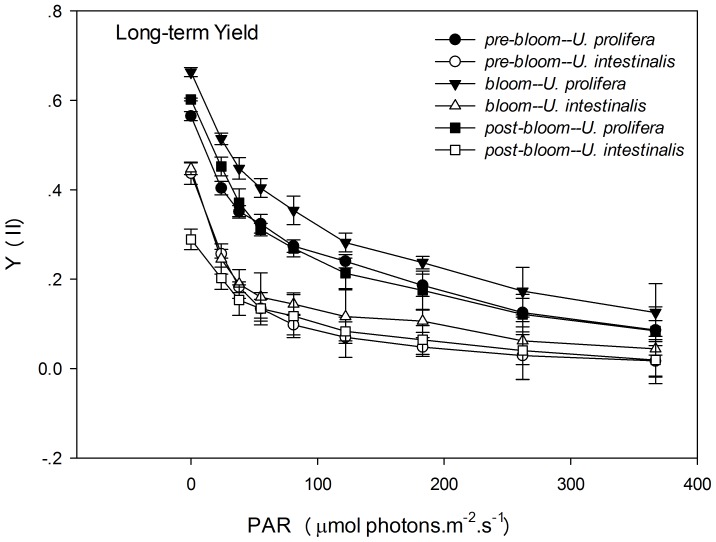
Mean effective quantum yield (Y (II)) of rapid light response curves (RLCs) of *U. prolifera* and *U. intestinalis*, obtained from long-term (7 d) culture, measured under different culture treatments: *pre-bloom*, *bloom*, and *post-bloom*. Values are means ± SD (n = 5). Different letters above bars indicate significantly different values (post-hoc, *P*<0.05).

Y (II) and rETR are two different indicators of rapid light respond curves (RLCs). These were measured to determine photosynthetic performance. Y (II) decreased with set light intensity gradient, and Y (II) of *U. prolifera* remained higher that the corresponding value for *U. intestinalis* at each level of light intensity in each experiment, especially in the long-term treatment (Figs. 5 and 6). Maximum Y (II) values measured at the beginning of the RLC were used to compare the effects of species and phase of development. Variations in the maximum Y (II) were similar to those of Fv/Fm, and the Y (II) of each phase was slightly smaller than that of Fv/Fm (Figs. 3, 4, 5, and 6). The maximum Y (II) of *U. prolifera* was significantly larger than that of *U. intestinalis* (Table 3, two-way ANOVA, *P*<0.0005). However, the effects of culture treatment (developing phase) were slight (Table 3, two-way ANOVA, *P*>0.05).

**Figure 7:**
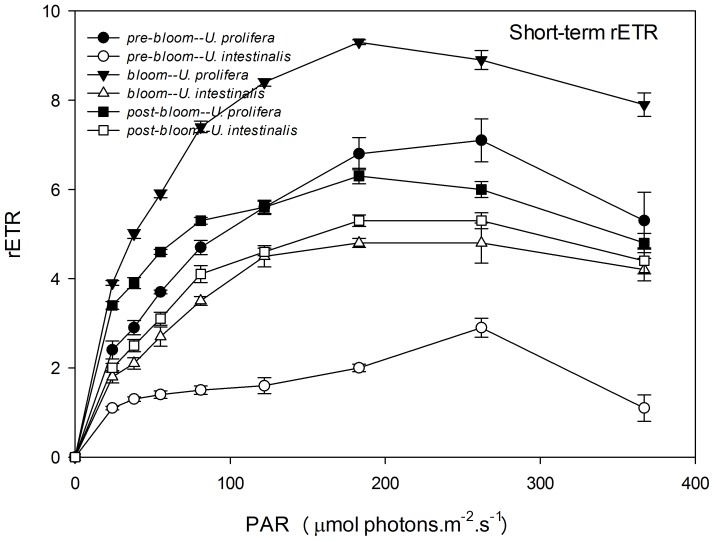
Mean relative electron transport rate (rETR) of rapid light response curves (RLCs) of *U. prolifera* and *U. intestinalis*, obtained from short-term (24 h) culture to each, measured under different culture treatments: *pre-bloom*, *bloom*, and *post-bloom*. Values are means ± SD (n = 5). Different letters above bars indicate significantly different values (post-hoc, *P*<0.05).

**Figure 8:**
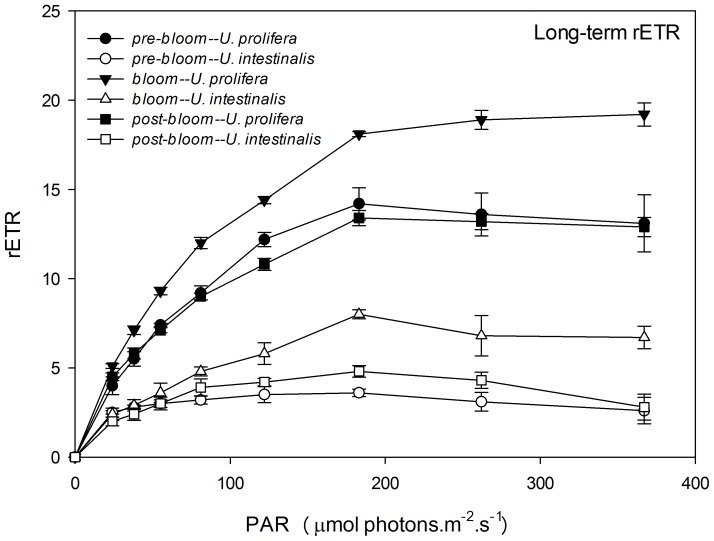
Mean relative electron transport rate (rETR) of rapid light response curves (RLCs) of *U. prolifera* and *U. intestinalis*, obtained from long-term (7d) culture, measured under different culture treatments: *pre-bloom*, *bloom*, and *post-bloom*. Values are mean ± SD (n = 5). Different letters above bars indicate significantly different values (post-hoc, *P*<0.05).

Like Y (II), rETR showed a clear pattern of photosynthetic performance, and RLCs appeared similar to a traditional oxygen-based photosynthesis-irradiance (P–I) curve (Figs. 7 and 8). It showed a linear rise until light became a limiting factor, followed by a plateau, where the photosynthetic pathway became limited. The convexities of the curve, both of *U. prolifera* and *U. intestinalis* thallus, were clearly higher in the long-term experiments than in short-term experiments (Figs. 7 and 8.). After RLCs were fitted, parameters α, rETRmax, and Ek were calculated to compare the exact photosynthetic performance. rETRmax and Ek showed significant differences with respect to species and phase of development (Tables 3 and 4, two-way ANOVA, *P*<0.0005), and α only showed significant differences across species but not phase of development (Table 4, post-hoc, *P*>0.05). These values also showed almost the same photosynthetic physiological status, like Fv/Fm and Y (II). These parameters of *U. prolifera* thallus were significantly more pronounced than those of *U. intestinalis* in both the short-term and long-term experiments. The maximum value appeared under the *bloom* culture conditions, as Fv/Fm did (Figs. 7 and 8, Table 4). The rETRmax and Ek of these two species showed a sharp increase from short-term to long-term under *pre-bloom* and *bloom* conditions but not under *post-bloom* conditions(Table 4).

**Table 4:** Mean parameters of the RLC in *U. prolifera* and *U. intestinalis* under different culture treatments: *pre-bloom*, *bloom*, and *post-bloom*.

Treatment	rETRmax		α		Ek	
Species	short-term	long-term	short-term	long-term	short-term	long-term
***pre-bloom***						
*U. prolifera*	6.43^b^ ±1.02	14.06^b^ ±1.96	0.11^ab^	0.20^a^	59^a^	70^b^
*U. intestinalis*	1.90^e^ ±0.68	3.19^d^ ±0.39	0.05^c^	0.19^a^	37^c^	17^d^
***Bloom***						
*U. prolifera*	8.75^a^ ±0.76	19.75^a^ ±3.17	0.20^a^	0.23^a^	44^b^	85^a^
*U. intestinalis*	4.70^d^ ±0.54	7.25^c^ ±1.19	0.08^b^	0.10^b^	57^a^	70^b^
***post-bloom***						
*U. prolifera*	5.68^c^ ±0.58	13.62^b^ ±1.91	0.19^a^	0.21^a^	30^d^	65^b^
*U. intestinalis*	5.04^cd^ ±0.54	4.06^d^ ±0.74	0.10^ab^	0.11^b^	52^ab^	36^c^

Samples were collected after short-term (24 h and long-term (7 d) culture. Different superscripts indicate significantly different values (*P*<0.05) as determined by post-hoc comparisons.

### 3. Lipid peroxidation

Biochemical parameters (lipid peroxidation, antioxidant system, and nitrate reductase) showed highly significant differences across treatments (Table 5, two-way ANOVA, *P*<0.01). Only GSH showed no difference in the combined effect of species and phase of development (F_2, 24_ = 0.115, *P* = 0.334).

**Table 5:** Results of two-way ANOVA on effects of culture treatments (developing phase) and species (species) on the biochemical assay results of total soluble protein (TSP), total antioxidant ability (T-AOC), malondialdehyde (MDA), superoxide dismutase (SOD), glutathione peroxidase (Gpx), glutathione (GSH), and nitrate reductase (NR).

Source	Dependent Variable	*df*	*F*-ratio	*p*-value
developing phase	TSP	2	172.275	0.000
	T-AOC	2	176.909	0.000
	MDA	2	12.540	0.000
	SOD	2	35.580	0.000
	Gpx	2	7.683	0.003
	GSH	2	9.858	0.001
	NR	2	72.649	0.000
Species	TSP	1	1090.687	0.000
	T-AOC	1	11.918	0.002
	MDA	1	14.868	0.001
	SOD	1	89.471	0.000
	Gpx	1	81.952	0.000
	GSH	1	8.612	0.007
	NR	1	39.766	0.000
developing phase × species	TSP	2	72.952	0.000
	T-AOC	2	8.499	0.002
	MDA	2	3.900	0.034
	SOD	2	15.924	0.000
	Gpx	2	14.646	0.000
	GSH	2	1.115	0.344
	NR	2	10.437	0.001
Error		24		

**Figure 9:**
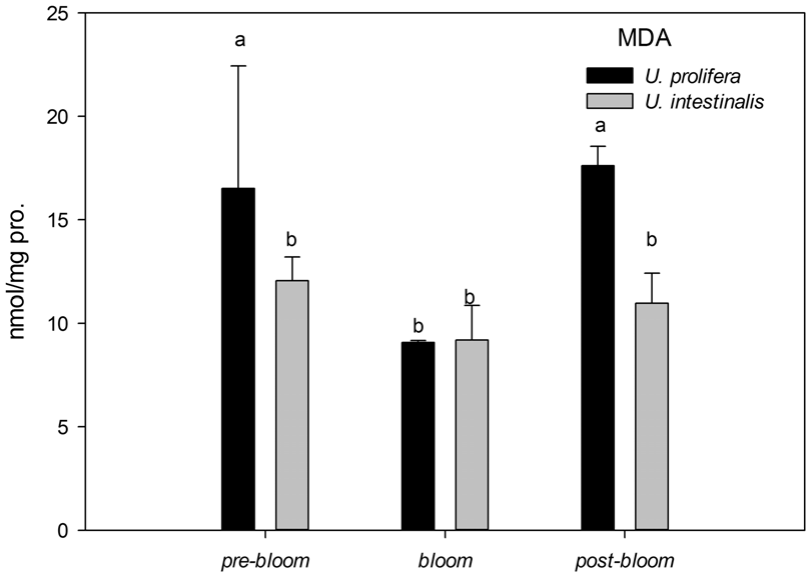
Mean lipid peroxidation studied as malondialdehyde (MDA) of *U. prolifera* and *U. intestinalis*, measured under different culture treatments: *pre-bloom*, *bloom*, and *post-bloom*. Values are means ± SD (n = 5). Different letters above bars indicate significantly different values (post-hoc, *P*<0.05).

MDA concentrations showed statistically significant increases in the *U. prolifera* only under *pre-bloom* and *post-bloom* conditions (Fig. 9, post-hoc, *P*<0.05), providing a measure of lipid oxidative damage of the treatments. This effect was slighter in *U. intestinalis* (Fig. 9, post-hoc, *P*>0.05), showing no difference across the three culture treatments.

### 4. Antioxidant system assays

**Figure 10:**
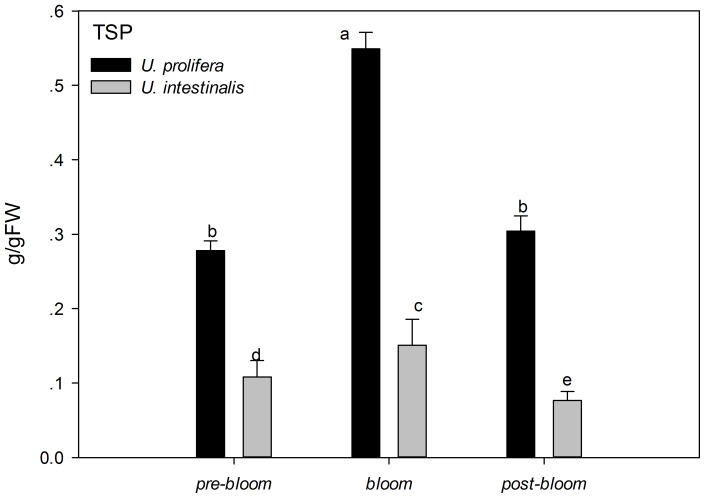
Mean total soluble protein (TSP) of *U. prolifera* and *U. intestinalis*, measured under different culture treatments: *pre-bloom*, *bloom*, and *post-bloom*. Values are means ± SD (n = 5). Different letters above bars indicate significantly different values (post-hoc, *P*<0.05).

**Figure 11:**
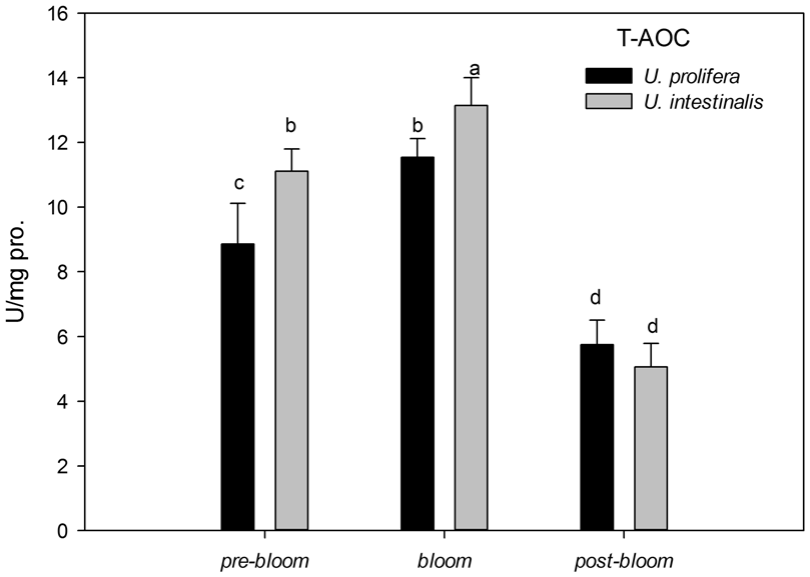
Mean total antioxidant ability (T-AOC) of *U. prolifera* and *U. intestinalis*, measured under different culture treatments: *pre-bloom*, *bloom*, and *post-bloom*. Values are means ± SD (n = 5). Different letters above bars indicate significantly different values (post-hoc, *P*<0.05).

Two-way ANOVA showed significantly higher TSP concentrations in *U. prolifera* than *U. intestinalis* (Table 5, *P*<0.0005), and the TSP of both species was significantly lower under *pre-bloom* and *post-bloom* culture treatments (Fig. 10, post-hoc, *P*<0.05). T-AOC as an estimator of total antioxidant capacity in defense system had a lower value in *U. prolifera* than *U. intestinalis*. It also showed a marked decrease under the *post-bloom* conditions (Fig. 11).

**Figure 12:**
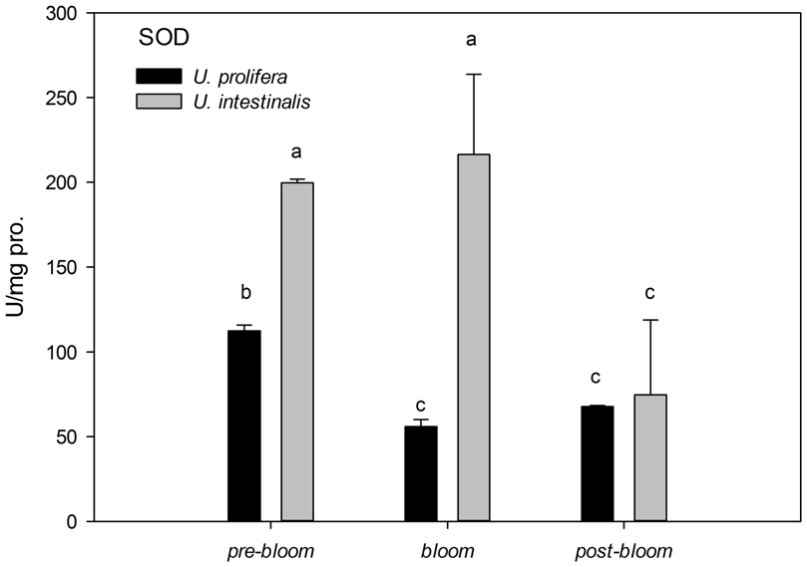
Mean activities of antioxidant enzymes superoxide dismutase (SOD) of *U. prolifera* and *U. intestinalis*, measured under different culture treatments: *pre-bloom*, *bloom* and *post-bloom*. Values are means ± SD (n = 5). Different letters indicate significantly different values (post-hoc, *P*<0.05).

**Figure 13:**
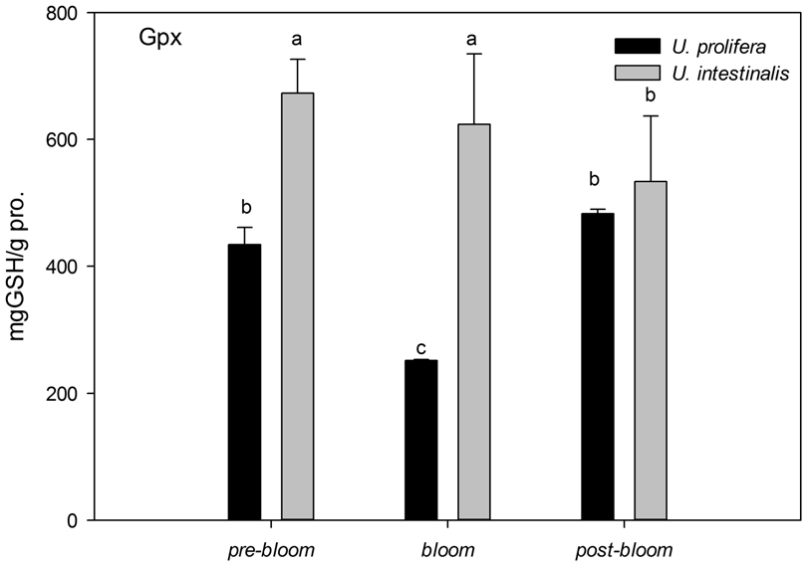
Mean activities of antioxidant enzymes glutathione peroxidase (Gpx) of *U. prolifera* and *U. intestinalis*, measured under different culture treatments: *pre-bloom*, *bloom*, and *post-bloom*. Values are means ± SD (n = 5). Different letters above bars indicate significantly different values (post-hoc, *P*<0.05).

The activities of SOD and Gpx in *U. prolifera* were much lower than in *U. intestinalis* under all culture treatments, but the difference under the *post-bloom* culture treatment was not significant (Figs. 12 and 13, post-hoc, *P*>0.05). *U. prolifera* under *pre-bloom* and *post-bloom* culture treatments trended to have higher antioxidant enzyme activities than under *bloom* conditions (Figs. 12 and 13), although this was not statistically significant for SOD under *post-bloom* culture treatment (post-hoc, *P*>0.05). However, *U. intestinalis* did not show such changes (post-hoc, *P*>0.05). These two enzymes were found to have more sensitive responses in *U. prolifera* than in *U. intestinalis* under all three sets of conditions.

**Figure 14.**
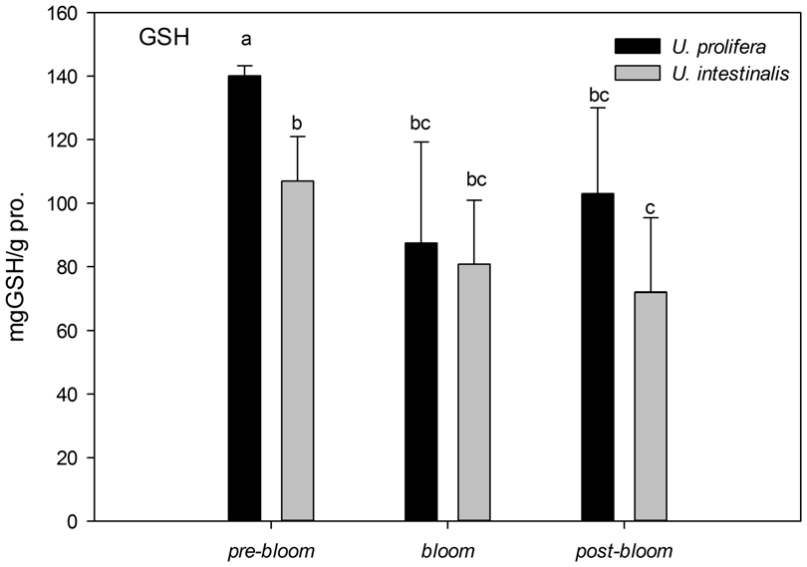
Mean activities of non-enzyme antioxidant glutathione (GSH) of *U. prolifera* and *U. intestinalis*, measured under different culture treatments: *pre-bloom*, *bloom*, and *post-bloom*. Values are means ± SD (n = 5). Different letters above bars indicate significantly different values (post-hoc, *P*<0.05).

The activity of GSH, as the major endogenous non-enzyme antioxidant, was higher in *U. prolifera* than in *U. intestinalis* (Fig. 14), especially under *pre-bloom* conditions (post-hoc, *P*<0.05). Like the antioxidant enzymes SOD and Gpx, GSH showed more activity in *U. prolifera* under *pre-bloom* and *post-bloom* conditions than under *bloom* conditions, but this was not true in *U. intestinalis*.

### 5. Nitrate reductase analysis

**Figure 15:**
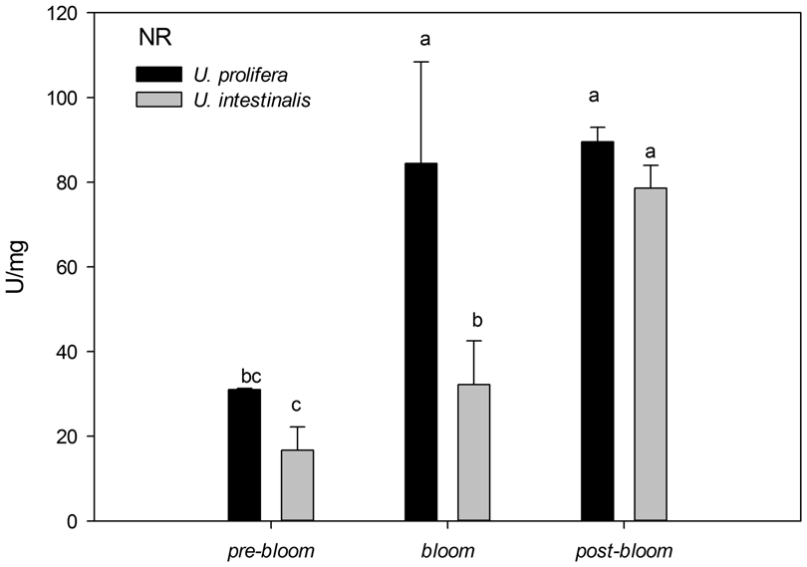
Mean activities of N-absorption related enzymes nitrate reductase (NR) of *U. prolifera* and *U. intestinalis*, measured under different culture treatments: *pre-bloom*, *bloom*, and *post-bloom*. Values are means ± SD (n = 5). Different letters above bars indicate significantly different values (post-hoc, *P*<0.05).

The activities of NR in these two species were significantly higher under *bloom* and *post-bloom* conditions than under *pre-bloom* conditions (Fig. 15). Under the *bloom* culture treatment, the activity of NR in *U. prolifera* was significantly higher than in *U. intestinalis*, with a mean of 84.41±24.00 U/mg in *U. prolifera* but only 32.24±10.28 U/mg in *U. intestinalis*.

## Discussion

Data from the field investigation showed that the changes of the environmental factors had a close relationship with the phases of the development of green tide. We have observed that the *U. prolifera* green tide undergoes consistent phases of development: It forms in May to late July and diminishes in late July to early September. These findings were almost exactly the same as those of previous studies [7], [15], [16], [30]. The present study is the first to correlate the phases of the annual macroalgal bloom with regular variations in environmental factors such as irradiance, temperature, and salinity. These results provided the basis for our subsequent laboratory experiments.

The methods used in this study provide an estimate of maximum physiological responses to single and combined effects of environmental factors and a means of replicating the natural process. The algae that cause coastal green tides live under a suite of changing coastal environmental conditions. Only when these factors are assessed experimentally can we begin to understand when and why algal blooms proliferate [21]. In most laboratory studies, a single environmental parameter is imposed and its effects on algal growth are analyzed. However, adverse abiotic factors are almost never present alone in natural habitats of benthic marine or estuarine algae [31]. For example, the high light stress caused by low tide is often accompanied by high temperature stress, and this combination may also induce osmotic disturbances. For this reason, we designed this special factorial laboratory experiment to analyze the single and combined effects of irradiance, temperature, and salinity on the physiological responses of co-occurring *U. prolifera* and *U. intestinalis*. We eliminated many abiotic stress factors that can interact synergistically within respective bloom periods and other inhibiting effects such as nutrient limitation and herbivory. Our results confirmed that *U. prolifera* and *U. intestinalis* differ in their physiological responses under different culture treatment. We proved the method successful in the laboratory.

### Capacity and plasticity of photosynthesis

The amount of solar radiation absorbed by a leaf is a function of the photosynthetic pigment content. Chlorophyll content can directly determine maximum potential photosynthesis [32]. Chl and Chla were significantly affected by species (Table 1), especially under *bloom* conditions. Significantly higher chlorophyll concentrations were observed in *U. prolifera* over *U. intestinalis* (Table 2). The same pattern was observed with Fv/Fm. In this way, the higher Chl and Chla concentrations contributed in part to higher rates of photosynthesis in *U. prolifera* under the *bloom* culture conditions. We also found the concentration of chlorophyll b to be more affected than chlorophyll a by experimental treatments both in the short-term and long-term experiments. We observed that the concentration of chlorophyll b in *U. prolifera* was much higher under short-term conditions than long-term conditions (Table 1). Increases in accessory pigments (e.g., Chl b) relative to antenna pigments (e.g., Chl a) increased the efficiency of photosynthesis by enhancing PS II conversion of light to chemical energy. This is why more photosynthesis takes place in *U. prolifera* during the bloom period than during other periods.

The pigments did not show pronounced changes in concentration with respect to culture treatment, especially under long-term conditions (Table 1). Similar results had been observed in marine green algae by Luo and Liu ([15], salinities stress effect vs *U. prolifera*) and by Bischof et al ([33], UV-B stress effect vs *U. lactuca*). However, the reason for this is still unknown. The slow response of pigments (chlorophylls) to stress relative to that of other physiological parameters may have contributed to these results. Pigment content usually takes several days to weeks to acclimate. This has been recognized in previous reports on *Ulva* [34].

After the chlorophyll fluorescence measurements, our key finding was that *U. prolifera* had a higher photosynthetic capacity than *U. intestinalis*. The ratio of dark-adapted Fv/Fm and maximum Y (II) in *U. prolifera* was significantly higher than that of *U. intestinalis*, especially under *bloom* conditions. This suggested that *U. prolifera* had greater photosynthetic efficiency. This conclusion is further evidenced by the observations of RLCs. *U. prolifera* showed a steadier and slower decline in Y (II) over the course of the RLCs than *U. intestinalis* did (Figs. 5 and 6). This implies that *U. prolifera* maintained a greater level of photosynthetic activity than *U. intestinalis*. This was reflected in substantially higher maximum rETR, α, and Ek. Thallus morphology may also explain some of the difference in photosynthetic efficiency. Previous studies found that thinner and more filamentous species have higher rates of photosynthesis than coarser, thicker species [35], [36]. Filamentous *U. prolifera* has a higher surface-volume ratio than tubular *U. intestinalis*. This plays an important role in influencing photosynthetic capacity. The higher photosynthetic capacity of *U. prolifera* largely contributes to its larger biomass and increases its ability to form green tides.

After 7 days of stress treatments, *U. intestinalis* demonstrated a pronounced tolerance to certain culture treatments. Slighter effects were observed with Fv/Fm and Y (II) responses (Figs. 3 and 4, Table 3). The maintenance of dark-adapted Fv/Fm and Y (II) by *U. intestinalis* suggested that it was less prone to photosystem damage than *U. prolifera*. *U. prolifera* was more susceptible to stress treatment than *U. intestinalis*, especially to the *post-bloom* culture stress conditions. In the short-term, *U. prolifera* showed a lower ratio of photolysis parameters to the sudden stress, and under the *pre-bloom* and *bloom* culture conditions, these parameters showed a sharp increase over long-term culture (7 days). However, under the *post-bloom* treatment, *U. prolifera* did not show higher Fv/Fm, rETRmax, or Ek values during short-term culture. This indicated that recovery of photosynthesis in both species was inhibited by combined environmental factors under *post-bloom* conditions. The thinner and more filamentous thallus morphology may also have partly contributed to this inhibition. This morphology exposes more surface area to stress. Our biochemical results provided another explanation, discussed below. In this way, the reason why green tides caused by *U. prolifera* diminish is that it cannot adapt to *post-bloom* stress with high SST, high PAR, and low SSS either in the laboratory or in the more complex *post-bloom* environment that occurs in the field. Judging by to its photosynthetic plasticity, *U. prolifera* has more capacity for photosynthesis under *pre-bloom* and *bloom* conditions, creating ephemeral blooms in the field.

### Different defense systems

Every species has its own ideal irradiance, temperature, and salinity. When any of these factors stray beyond a suitable range, photosynthesis and growth become limited. Culture conditions that incorporate combined environmental factors, as in this study, can be divided into two parts: suitable conditions (*bloom*) and stress conditions (*pre-bloom* with low SST, low PAR, and high SSS and *post-bloom* with high SST, high PAR, and low SSS). Many case studies on species of the genus *Ulva* have used the production of reactive oxygen species to indicate the influence of environmental stress on their growth [37]. Results have indicated that PSII activity and antioxidant systems in these *Ulva* species are sensitive to stress conditions [13], [15], [38].

In the present study, oxidative stress was induced when alga samples were exposed to environmental stress conditions. This phenomenon was more pronounced in *U. prolifera*, as directly indicated by increases in MDA content as the parameter of lipid peroxidation and indirectly indicated by increases in the activity levels of antioxidant systems. The concentration of MDA in *U. prolifera* was significantly higher under stress culture treatment conditions, which indicates that the normal metabolism of this alga may be seriously disrupted by ROS through oxidative damage to lipids, protein, and nucleic acids. Meanwhile, the antioxidant system of this species showed the same pattern: The activities and concentrations of antioxidant enzymes (SOD, Gpx) and non-enzyme antioxidants (GSH) increased in response to damage. *U. prolifera* showed significantly lower SOD and GSH levels under *post-bloom* conditions than under *pre-bloom* conditions (Figs. 12–14). This suggests that *U. prolifera* might experience greater damage and so produce more ROS than it can easily remove. However, the responses of *U. intestinalis* did not differ significantly when it was exposed to the same stress conditions. Both species showed comparable levels of antioxidant enzymes activity. It can be said that *U. prolifera* is more susceptible to environmental stress than *U. intestinalis* is. A correlation between antioxidant system response and stress tolerance has been reported in other species of green algae [37], [39], [40]. When Choo et al. compared oxidative stress tolerance in two green algae *Cladophora glomerata* and *U. ahlneriana*, they saw similar results [39]. We have also classified *U. prolifera* as a more stress-susceptible species and *U. intestinalis* as a more stress-tolerant species.

The traits of the different defense systems of these two co-occurring species are correlated with their different natural ecological processes. The increased level of scavenging activity (T-AOC, SOD, Gpx, and GSH) corresponded to the higher degree of lipid peroxidation (MDA) in *U. prolifera* observed in this study (Figs. 9–14). Under the *post-bloom* stress culture conditions, the value of scavenging activity (T-AOC, SOD, and GSH) was significantly lower than under *pre-bloom* stress conditions (post-hoc, *P*<0.05). The comparatively low level of PS II activity in *U. prolifera* following stress treatments implies the generation of enhanced ROS, because previous study had shown blockage of photosynthetic electron transport, which would lead to overproduction of ROS [41]. In *U. prolifera*, the lowest pigment concentration was observed under the *post-bloom* culture treatment in both short- and long-term scales, but this was not true of *U. intestinalis* (Table 2). The Fv/Fm of *U. prolifera* did not recover or increase under *post-bloom* conditions in either the short- or the long-term, so we concluded that insufficient antioxidative activity in *U. prolifera* under the *post-bloom* culture treatment conditions cannot prevent or reverse ROS damage. In this way, the ephemeral bloom of *U. prolifera* and the persistence of *U. intestinalis* can be explained.

### Other factors affecting the ecological success of *U. prolifera*

Meteorological events can contribute to the blooming and fading of green tide in shallow coastal areas such as the one investigated here. Rainfall was rare during winter, moderate in April through June, and showed a sudden sharp increase from July to September (Fig. 1a). Moderate, increasing rainfall causes increased input of particulate suspended materials and nutrients into the water column. This, in turn, contributes to decreased salinity and increased nutrient levels (DIN) (Fig. 1a). DIN was negatively correlated with salinity in the Yellow Sea green tide outbreak area. This indicates that the source of nutrients is rainfall or rainfall-induced runoff [42], [43]. This situation should favor *U. prolifera* bloom formation because *U. prolifera* has a higher nitrogen utilization capacity than *U. intestinalis* does (Fig. 15). Typhoons co-appeared with the sudden increase in rainfall from mid-July through September (Fig. 1a). These caused harsh environmental changes, with temperatures soaring and rainfall causing sudden drops in salinity. These typhoons caused environmental stresses that can break the weaker defense system of *U. prolifera* and diminish the severity of a green tide.

Another biochemical analysis showed that the concentration of total soluble protein (TSP) in *U. prolifera* was significantly higher than in *U. intestinalis*, and this value decreased under the stress conditions. TSP include photosynthetic proteins (such as rubisco, D_1_ proterin), nutrient utilization absorption related proteins (such as NR), proteins related to the defense system (such as antioxidant enzyme and HSP), and so on. Among these proteins, only NR was clearly found to contribute to TSP in this study. This compound appears in very high concentrations, especially under the *bloom* culture treatment.

*Ulva* species are known to have a high nitrogen requirements for optimal growth [44]. However, nitrogen is the limiting factor in the open ocean of the Yellow Sea [9]. During our field investigation, we found that levels of dissolved inorganic nitrogen (DIN) decreased from April to July (ranging from 21.9 to 8.2 μmol/L). This may favor *U. prolifera* over *U. intestinalis* from May to June. In this way, higher nitrogen utilization capacity indicates higher biomass. Therefore, *U. prolifera*, unlike *U. intestinalis*, can form blooms when the other factors (like the environment) are suitable.

Photosynthetic protein may also have a correlation with TSP. This is because the concentration of TSP has a pattern similar to that of Fv/Fm, which was here used as indicator of photosynthetic capacity. The decrease may also be affected by ROS. ROS has recently been identified as an important component in certain signal transduction pathways [41]. For example, ROS can cause the inhibition of gene expression of key proteins involved in photosynthesis (e.g. *rbc*L, *rbc*S encoding for the large and small rubisco subunit, and *psb*A encoding for the D_1_ protein in PSII) [45]. As signaling molecules, they can be used to induce heat shock proteins (HSPs) [46]. However, the exact reason for difference in TSP values between *U. prolifera* and *U. intestinalis* stresses has remained unclear. Linking physiological responses to stress with their genetic bases, particularly using current genomic and proteomic approaches, would certainly improve the understanding of stress responses (like TSP values) of these macroalgal species. Few studies on the factors that regulate genes (e.g. antioxidant enzymes, photosynthetic proteins, and heat shock proteins) in macroalgae exposed to stress are available. In our subsequent experiments, quantitative real time RT-PCR was found to be an efficient means of performing comparative transcriptional analysis. This technique will be used to investigate whether stress responses occur in *U. prolifera* and determine how the defense systems of these macroalgal species are regulated under stress conditions. These qRT-PCR results will further confirm the findings reported in the present study.

From the present study we can draw three key **conclusions**: (1) The method used in this study provides a maximum estimate of the physiological responses to environmental changes, maximum replication of natural processes, and comparison of results found in the laboratory. This allows us to develop explanations of how *U. prolifera* can form green tides in the Yellow Sea under natural conditions. (2) Differences of ecophysiological traits in photosynthesis and defense system of these two co-occurring *Ulva* macroalgae under combined abiotic conditions correlated with their different natural processes: *U. prolifera* was found to be more sensitive to dynamic environments, especially to harsh or changing environmental conditions, characterizing their purely opportunistic strategy. *U. intestinalis* is more stable, but this is largely due to the stress tolerance provided by antioxidant system. (3) The ecological success of *U. prolifera* is inextricably linked to its higher capacity, its plasticity, and its nutrient absorption and assimilation systems. Once conditions become favorable, *U. prolifera* seems to be able to successfully compete with *U. intestinalis* for nutrients and space.
